# PIM2, lactate, and trauma score to predict mortality in critically ill pediatric trauma patients

**DOI:** 10.1016/j.jped.2026.101509

**Published:** 2026-02-17

**Authors:** Luciana G. Barcellos, Fernanda M. Rubin, Ana Paula P. da Silva, Júlia L. Vieira, Luciane G. da Cunha, Lucinara V. Enéas Machado, Geniara da S. Conrado, Cristian T. Tonial

**Affiliations:** a Hospital de Pronto Socorro de Porto Alegre, Pediatric Trauma Intensive Care Unit, Porto Alegre, RS, Brazil; b Hospital de Clínicas de Porto Alegre (HCPA), Federal University of Rio Grande do Sul (UFRGS), Porto Alegre, RS, Brazil

**Keywords:** Wounds and injuries, Pediatrics, Prognosis, Lactic acid, Intensive Care Units, Pediatric, Trauma Centers

## Abstract

**Objective:**

To evaluate the prognostic performance of the Pediatric Index of Mortality 2 (PIM2), serum lactate, and Pediatric Trauma Score (PTS) for mortality in a large case series of critically ill pediatric trauma patients admitted to a specialized PICU in Brazil.

**Methods:**

Retrospective case series conducted in the Pediatric Intensive Care Unit of a tertiary trauma hospital in Brazil. All trauma patients aged 1 month to 18 years admitted between March 2018 and March 2025 and hospitalized for >24 h were eligible (*n* = 1495). Demographic, clinical, and laboratory data were collected, including PIM2, initial lactate, and PTS. The primary outcome was all-cause PICU mortality.

**Results:**

Death occurred in 1.5% of patients. ROC curve analysis was performed in 620 patients with complete data for the three markers. Areas under the curve (AUCs) were: PIM2, 0.93 (95% CI, 0.91–0.95); lactate, 0.86 (95% CI, 0.83–0.88); and PTS, 0.82 (95% CI, 0.79–0.85). In univariable logistic regression, all markers were independently associated with mortality. A 10-fold increase in PIM2 and lactate raised death odds by 36-fold and 226-fold, respectively, while each point increase in PTS reduced odds by 34.6%. In the multivariable model, PIM2 and lactate remained significant predictors.

**Conclusions:**

PIM2 and lactate remained independently associated with mortality after mutual adjustment in pediatric trauma patients admitted to a specialized PICU. PTS, while valuable for prehospital triage, added little once intensive care was initiated.

## Introduction

Brazil has long been recognized as a country with high levels of violence, reflected in World Health Organization statistics. In 2021, trauma was the leading cause of death among children in the country, including interpersonal violence, traffic accidents, and drownings. Among adolescents aged 15 to 19 years, self-harm, collective violence, and legal intervention were also prominent [1].

To meet the demand for specialized trauma care, the city government of Porto Alegre – the capital of Brazil’s southernmost state — established the nation’s first pediatric intensive care unit (PICU) dedicated exclusively to trauma in the early 2000s. Since then, this 8-bed unit has played a vital role in delivering critical care to pediatric trauma victims.

Prognostic markers help identify patients needing intensive care, guide resource allocation and predict deterioration. Ideally, they should be inexpensive, available in PICUs and simple to use. Among the most widely used and accessible are serum lactate, the Pediatric Index of Mortality 2 (PIM2), and the Pediatric Trauma Score (PTS).

Serum lactate elevation in trauma patients is multifactorial and carries prognostic significance. It may result from increased catecholamine levels—especially epinephrine — released in response to severe injury, cardiogenic or hemorrhagic shock, or regional hypoxia due to microcirculatory dysfunction, all of which contribute to hyperlactatemia [2]. PIM2 is a well-established mortality prediction score, routinely used in general PICUs worldwide, and calculated from clinical data within the first 24 h of admission [3]. The PTS, developed in the 1980s, is a clinical score used during initial trauma assessment, incorporating vital signs, anthropometric data, and clinical parameters to estimate mortality risk and support triage and transfer decisions [4]. Although each marker shows prognostic value, they have never been jointly assessed in pediatric trauma.

The aim of this study is to describe the clinical and demographic profile of pediatric trauma patients admitted over a 7-year period to a specialized trauma PICU in Brazil, and to investigate the prognostic value of serum lactate, PIM2, and PTS when analyzed together within a large and representative case series.

## Methods

This retrospective case series study was conducted in the Pediatric Intensive Care Unit (PICU) of the Hospital de Pronto Socorro de Porto Alegre (HPS) after approval by the Institutional Review Board, under Ethics Approval Certificate number CAAE 79,319,624.9.0000.5338. HPS is a tertiary-level teaching hospital dedicated to emergency and trauma care, operating 24 h a day with on-call coverage across all medical specialties. It is the main trauma hospital in the state of Rio Grande do Sul (population of 11 million).

Access to care is provided entirely through the Brazilian Unified Health System (SUS), a government-funded universal health care system that offers physician and hospital services free of charge, with no copayments or user fees. The hospital operates a single, integrated emergency department for both adults and children. However, the majority of pediatric patients are referred through the city’s prehospital emergency system or transferred from other institutions within the capital or the state. HPS has a total of 139 inpatient beds, including 28 intensive care unit (ICU) beds. The PICU comprises 8 beds dedicated to medical and surgical pediatric patients ranging in age from 0 to 18 years. The unit admits approximately 220 patients per year.

Eligible patients were all those admitted to the PICU for trauma between March 15, 2018, and March 21, 2025, who remained hospitalized > 24 h. No exclusion criteria were applied. This period was chosen because electronic records were unavailable before March 2018. The authors reviewed all eligible records, extracting clinical, laboratory, and vital sign data.

Collected variables included demographics (age, sex, weight, admission date/time, trauma location and type), prehospital care, suspected non-accidental trauma (NAT) or suicide attempt, Pediatric Index of Mortality 2 (PIM2), Pediatric Trauma Score (PTS), and Pediatric Glasgow Coma Scale [3, 4, 5].

Laboratory data comprised serum lactate (mmol/L), defined as the first measurement after admission. The authors also recorded use of mechanical ventilation (MV), ventilator-free days, tracheostomy, vasoactive drugs, blood transfusion, nosocomial infection (new infection during hospitalization), enteral/parenteral nutrition, PICU and hospital length of stay, complicated outcomes, brain death, and discharge status, including death.

Duration of mechanical ventilation was calculated using the ventilator-free days approach proposed by Schoenfeld et al., with the formula: 28 minus days on MV. Patients who died or required MV > 28 days were assigned 0, while those not requiring MV were assigned 28 [6]. A complicated outcome was defined as at least one of the following: MV > 7 days, vasoactive drug use, or death.

For statistical analysis, categorical variables were expressed as counts and percentages, while continuous variables were described as means with standard deviations (SD) or medians with interquartile ranges (IQR), depending on data distribution. Categorical variables were compared using the chi-square test or Fisher’s exact test, as appropriate, with Bonferroni correction applied for multiple comparisons. For continuous variables, Student’s *t*-test was used when data were normally distributed (based upon Kolmogorov-Smirnov and Shapiro-Wilk tests), and the Mann-Whitney *U* test was applied for nonparametric data.

To assess the discriminative ability of PIM2, lactate, and PTS for mortality, areas under the receiver operating characteristic (ROC) curves were calculated and compared using the method described by DeLong et al. [7]. These three markers were selected due to their rapid availability, low cost, and accessibility in most trauma units. Logarithmic transformation was applied to PIM2 and lactate values prior to analysis due to their skewed distribution.

Univariable and multivariable logistic regression analyses were performed to evaluate the association of each marker with mortality. Statistical analyses were conducted using SPSS version 17.0 (IBM SPSS Statistics, Armonk, NY) and MedCalc version 15.8 (MedCalc Software BVBA, Ostend, Belgium). A p value of < 0.05 was considered statistically significant.

## Results

During the study period, 1495 patients aged between 0 and 18 years were included. Of these, 615 (41.1 %) were transferred from the metropolitan region, 480 (32.1 %) from other areas of the state, and 400 (26.8 %) from the city of Porto Alegre. The annual distribution of admissions remained consistent throughout the study period, with no relevant quantitative changes observed during the COVID-19 pandemic. Similarly, no seasonal variation was noted in the distribution of admissions across different times of the year, although the catchment area shows four distinct seasons, being located in a subtropical climate.

Most of the patients admitted were male (61.5 %), and the median age was 51 months (IQR 18–107). Regarding the location where the trauma occurred, the majority of cases took place in the patient’s home (887 cases, 59.3 %), while only 29 events (1.9 %) occurred at school. Prehospital care at the scene of the accident was provided in just 17.3 % of cases. The most common types of trauma were scald injuries (24.4 %), falls from height (21.5 %), pedestrian traffic accidents (12.2 %), and motor vehicle collisions (9.5 %). Regarding the main trauma diagnosis, traumatic brain injury (38 %) and burns (33.8 %) were the most prevalent. Demographic data, severity scores, prognostic markers, trauma characteristics, and overall outcomes comparing survivors and non-survivors are presented in Table 1.

**Table 1:** Demographics, Trauma Profiles, and Outcomes in Survivors and Non-survivors Admitted to the Pediatric Trauma ICU Over a 7-Year Period.

**Variable**	**All cases (*n*=1495)**	**Survivors** **(*n*=1473)**	**Non-survivors** **(*n*=22)**	p-value*
**Sex, n (%)** Male Female	920 (61.5) 575 (38.5)	907 (61.6) 566 (38.4)	13 (59.1) 9 (40.9)	0.82
**Age in months, md (IQR)**	51 (18–107)	51 (18–107)	50 (18.2–124.5)	0.78
**Age group, n (%)** 0–6 years 6–12 years Over 12 years	894 (59.8) 407 (27.2) 194 (13)	880 (59.7) 403 (27.4) 190 (12.9)	14 (63.6) 4 (18.2) 4 (18.2)	0.55
**Weight in kg, md (IQR)**	18 (12–30)	18 (12–30)	20,5 (12–35.2)	0.46
**Year of injury, n (%)** 2018 2019 2020 2021 2022 2023 2024 2025	190 (12.7) 215 (14.4) 197 (13.2) 220 (14.7) 201 (13.4) 221 (14.8) 225 (15.1) 26 (1.7)	188 (12.8) 211 (14.3) 196 (13.3) 217 (14.7) 198 (13.4) 215 (14.6) 223 (15.1) 25 (1.7)	2 (9.1) 4 (18.2) 1 (4.5) 3 (13.6) 3 (13.6) 6 (27.3) 2 (9.1) 1 (4.5)	0.58
**Season, n (%)** Summer Autumn Winter Spring	401 (26.8) 361 (24.1) 359 (24) 374 (25)	395 (26.8) 355 (24.1) 351 (23.8) 372 (25.3)	6 (27.3) 6 (27.3) 8 (36.4) 2 (9.1)	0.28
**City of origin, n (%)** Capital Metropolitan Region Countryside	400 (26.8) 615 (41.1) 480 (32.1)	397 (27) 605 (41.1) 471 (32)	3 (13.6) 10 (45.5) 9 (40.9)	0.35
**Time of injury, n (%)** 0–5:59 am 6–11:59 am 12–5:59 pm 6–11:59 pm Unknown	67 (4.5) 253 (16.9) 629 (42.1) 512 (34.2) 34 (2.3)	66 (4.5) 248 (16.8) 620 (42.1) 506 (34.4) 33 (2.2)	1 (4.5) 5 (22.7) 9 (40.9) 6 (27.3) 1 (4.5)	0.86
**Location of trauma, n (%)** Home Relative´s or friend´s home School Public roads Other Unknown or not applicable	887 (59.3) 76 (5.1) 29 (1.9) 425 (28.4) 56 (3.7) 22 (1.5)	877 (59.5) 75 (5.1) 29 (2) 419 (28.4) 52 (3.5) 21 (1.4)	10 (45.5) 1 (4.5) 0 (0) 6 (27.3) 4 (18.2) 1 (4.5)	0.01
**Prehospital care, n (%)** Yes No Unknown	258 (17.3) 1160 (77.6) 75 (5.2)	249 (16.9) 1149 (78) 75 (5.1)	9 (40.9) 11 (50) 2 (9.1)	< 0.01
**Pediatric Trauma Score, md (IQR)**	8 (6–10)	8 (6–10)	2 (0–4)	< 0.01
**PIM 2%, md (IQR)**	0.8 (0.8–1.2)	0.8 (0.8–1.1)	39.1 (14.2–80.3)	< 0.01
**Lactate in mmol/L, md (IQR)**	1.7 (1.1–2.5)	1.7 (1.1–2.4)	5.5 (3.2–9.2)	< 0.01
**Pediatric Trauma Score < 8, n (%)** Yes No	501 (33.5) 994 (66.5)	479 (32.5) 994 (67.5)	22 (100) 0 (0)	< 0.01
**GCS, md (IQR)^a^**	15 (14–15)	15 (14–15)	3 (3–6)	< 0.01
**GCS by category, n (%)^b^** ≤ 8 9–12 13–15 Not measurable	46 (7.6) 38 (6.3) 437 (72.4) 82 (13.7)	43 (7.2) 37 (6.2) 436 (73.2) 80 (13.4)	3 (42.9) 1 (14.3) 1 (14.3) 2 (28.6)	< 0.01
**Type of Trauma, n (%)** Pedestrian struck Motor Vehicle collision Fall from height Gunshot wound Assault Scald injury Burn (flame) Electric shock Bicycle fall Bite or scratch Venomous animal injury (including spider) Exogenous poisoning Object falling on the child Drowning Horse kick Sharp force injury Hanging Crush injury Chemical burn Foreign body aspiration Others Unknown	183 (12.2) 142 (9.5) 321 (21.5) 37 (2.5) 20 (1.3) 365 (24.4) 122 (8.2) 22 (1.5) 51 (3.4) 22 (1.5) 16 (1.1) 34 (2.3) 49 (3.3) 29 (1.9) 18 (1.2) 9 (0.6) 5 (0.3) 3 (0.2) 3 (0.2) 4 (0.3) 32 (2.1) 8 (0.5)	181 (12.3) 139 (9.4) 321 (21.8) 35 (2.4) 20 (1.4) 365 (24.8) 117 (7.9) 22 (1.5) 51 (3.5) 22 (1.5) 16 (1.1) 34 (2.3) 49 (3.3) 22 (1.5) 18 (1.2) 9 (0.6) 3 (0.2) 3 (0.2) 3 (0.2) 3 (0.2) 32 (2.2) 8 (0.5)	2 (9.1) 3 (13.6) 0(0) 2 (9.1) 0 (0) 0 (0) 5 (22.7) 0 (0) 0 (0) 0 (0) 0 (0) 0 (0) 0 (0) 7 (31.8) 0 (0) 0 (0) 2 (9.1) 0 (0) 0 (0) 1 (4.5) 0 (0) 0 (0)	< 0.01
**Main injury type, n (%)** Traumatic brain injury Facial trauma Spinal cord injury Thoracic trauma Hepatic abdominal trauma Splenic abdominal trauma Renal abdominal trauma Hollow abdominal trauma Pancreatic abdominal trauma Genitourinary trauma Other abdominal trauma Skin trauma or scalping Musculoskeletal trauma Burn Drowning Exogenous poisoning Venomous animal injury (including spider) Electric shock Hypoxic-ischemic injury Medical (non-trauma) admission Ocular trauma Pelvic trauma Others	568 (38) 42 (2.8) 4 (0.3) 40 (2.7) 27 (1.8) 27 (1.8) 10 (0.7) 18 (1.2) 10 (0.7) 10 (0.7) 7 (0.5) 7 (0.5) 95 (6.4) 505 (33.8) 15 (1) 34 (2.3) 16 (1.1) 5 (0.3) 24 (1.6) 6 (0.4) 11 (0.7) 3 (0.2) 11 (0.7)	561 (38.1) 42 (2.9) 4 (0.3) 40 (2.7) 27 (1.8) 27 (1.8) 10 (0.7) 18 (1.2) 10 (0.7) 10 (0.7) 7 (0.5) 7 (0.5) 94 (6.4) 500 (33.9) 14 (1) 34 (2.3) 16 (1.1) 5 (0.3) 16 (1.1) 6 (0.4) 11 (0.7) 3 (0.2) 11 (0.7)	7 (31.8) 0 (0) 0 (0) 0 (0) 0 (0) 0 (0) 0 (0) 0 (0) 0 (0) 0 (0) 0 (0) 0 (0) 1 (4.5) 5 (22.7) 1 (4.5) 0 (0) 0 (0) 0 (0) 8 (36.4) 0 (0) 0 (0) 0 (0) 0 (0)	< 0.01
**Suicide attempt** Yes No	19 (1.3) 1476 (98.7)	17 (1.2) 1456 (98.8)	2 (9.1) 20 (90.9)	0.03
**Suspected non-accidental trauma** Yes No Unknown	32 (2.1) 1461 (97.7) 2 (0.1)	31 (2.1) 1440 (97.8) 2 (0.1)	1 (4.5) 21 (95.5) 0 (0)	0.72
**Mechanical ventilation** Yes No	279 (18.7) 1216 (81.3)	257 (17.4) 1216 (82.6)	22 (100) 0 (0)	< 0.01
**Ventilator-free days, md (IQR)^c^**	25 (21–27)	—	—	—
**Tracheostomy** Yes No	15 (1) 1480 (99)	14 (1) 1459 (99)	1 (4.5) 21 (95.5)	0.20
**Vasoactive drugs** Yes No	145 (9.7) 1350 (90.3)	123 (8.4) 1350 (91.6)	22 (100) 0 (0)	< 0.01
**Blood transfusion** Yes No	240 (16.1) 1254 (83.9)	225 (15.3) 1248 (84.7)	15 (68.2) 6 (31.8)	< 0.01
**Nosocomial infection (after 72 h of hospitalization)** Yes No	212 (14.2) 1283 (85.8)	207 (14.1) 1266 (85.9)	5 (22.7) 17 (77.3)	0.22
**Tube feeding** Yes No	369 (24.7) 1126 (75.3)	359 (24.4) 1114 (75.6)	10 (45.5) 12 (54.5)	0.04
**Use of TPN** **Yes** No Unknown	13 (0.9) 1481 (99.1) 1 (0.1)	11 (0.7) 1461 (99.2) 1 (0.1)	2 (9.1) 20 (90.9) 0 (0)	< 0.01
**PICU length of stay in days, md (IQR)**	5 (2–10)	5 (2–10)	2 (1–9,7)	0.05
**PICU stay > 7 days, n (%)** Yes No	484 (32.4) 1011 (67.6)	478 (32.5) 995 (67.5)	6 (27.3) 16 (72.7)	0.81
**Hospital length of stay in days, md (IQR)**	6 (3–14)	6 (3–14)	2.5 (1–12)	0.01
**Hospital stay > 14 days, n (%)** Yes No	371 (24.8) 1124 (75.2)	367 (24.9) 1106 (75.1)	4 (18.2) 18 (81.8)	0.62
**Complicated outcome^d^, n (%)** Yes No	157 (10.5) 1338 (89.5)	—	—	—
**Diagnosis of brain death, n (%)** Yes No	9 (0.6) 1486 (99.4)	—	—	—
**Discharge disposition, n (%)** Ward Home Transfer to another hospital Shelter or institution Death	651 (43.5) 713 (47.7) 105 (7) 4 (0.3) 22 (1.5)	—	—	—

PICU, Pediatric Intensive Care Unit; TPN, Total Parenteral Nutrition; PIM2, Pediatric Index of Mortality 2; GCS, Glasgow Coma Scale; md, median; IQR, interquartile range.

^a^ Data available for 521 patients.

^b^ Data available for 603 patients.

^c^ Ventilator-free days = 28 – (days on MV). A value of zero was assigned to patients who died.

^d^ Complicated outcome includes patients who died, required mechanical ventilation for >7 days, or received vasoactive drugs.

*Considered statistically significant if *p* < 0.05. Chi-square or Fisher’s exact test was used for categorical variables, as appropriate. For non-parametric continuous variables, the Mann-Whitney U test was applied.

When stratified by age group, children aged 0 to 6 years were more frequently involved in domestic accidents (*p* < 0.01), with scald injuries being particularly prevalent (36.6 %). After age 6, pedestrian and motor vehicle accidents were more common (*p* < 0.01). No significant differences in mortality were observed across age groups (*p* = 0.55). These findings are presented in Supplemental Material 1.

All prognostic markers showed statistically significant differences between survivors and non-survivors: the Pediatric Trauma Score had a median of 8 (IQR, 6–10) vs. 2 (IQR, 0–4), serum lactate in mmol/L was 1.7 (IQR, 1.1–2.4) vs. 5.5 (IQR, 3.2–9.2), PIM2 predicted mortality was 0.8 % (IQR, 0.8–1.1) vs. 39.1 % (IQR, 14.2–80.3), and the Pediatric Glasgow Coma Scale score was 15 (IQR, 14–15) vs. 3 (IQR, 3–6), respectively (*p* < 0.01 for all comparisons). The latter was not included in additional analyses due to its restriction to patients with head trauma.

Regarding patient outcomes, the median PICU length of stay was 5 days (IQR, 2–10), with 32.4 % of patients remaining in the unit for >7 days. Approximately one-quarter of the case series had a total hospital stay exceeding 14 days. A complicated outcome occurred in 10.5 % of cases. Nine patients (0.6 %) were diagnosed with brain death, and the overall case-fatality rate was 1.5 %, with no significant differences across age groups.

**Figure 1:**
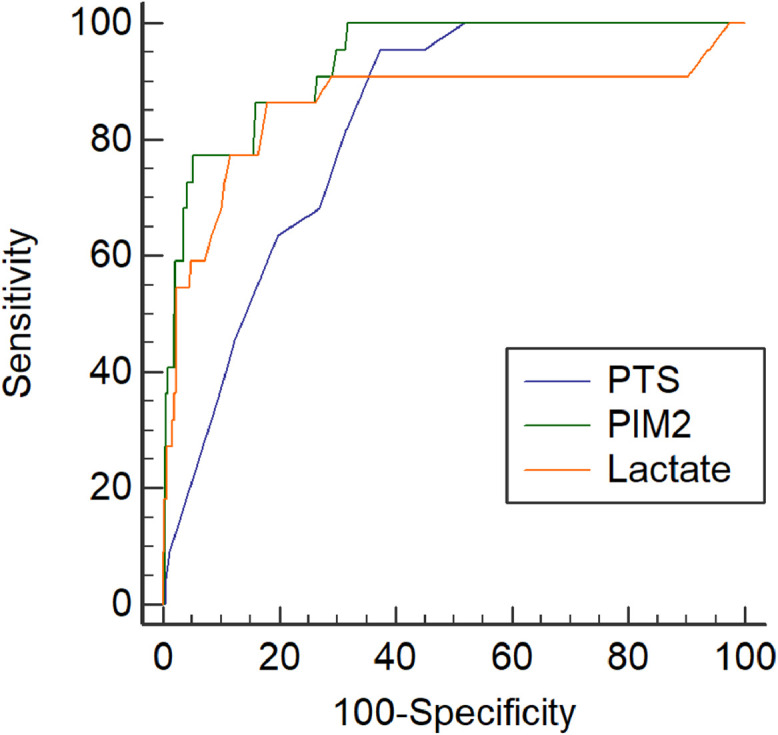
Receiver operating characteristic (ROC) curves for Pediatric Index of Mortality 2 (PIM2), lactate and Pediatric Trauma Score (PTS) in predicting mortality. The area under the curve (AUC) for PIM2 was 0.93 (95 % CI, 0.91–0.95), for lactate 0.86 (95 % CI, 0.83–0.88), and for PTS 0.82 (95 % CI, 0.79–0.85), indicating good discriminative performance of all parameters for the outcome of death.

The authors evaluated the discriminative ability for mortality using ROC curve analysis in the 620 patients for whom all three prognostic markers were available. All deaths occurred within this subgroup. The results are shown in Figure 1. The area under the curve (AUC) for PIM2 was 0.93 (95 % CI, 0.91–0.95), for serum lactate 0.86 (95 % CI, 0.83–0.88), and for the Pediatric Trauma Score 0.82 (95 % CI, 0.79–0.85). When the curves were compared using the DeLong et al. method (1988), PIM2 demonstrated superior performance compared to the Pediatric Trauma Score (*p* < 0.01). No statistically significant differences were found between PIM2 and lactate, or between lactate and the Pediatric Trauma Score.

Univariable logistic regression of the prognostic markers showed that, when analyzed separately, all three variables were strongly and independently associated with increased mortality risk. A 10-fold increase in PIM2 and serum lactate values was associated with a 36-fold and 226-fold increase in the odds of death, respectively. Conversely, each 1-point increase in the Pediatric Trauma Score was associated with a 34.6 % reduction in mortality risk.

In the multivariable logistic regression model including all three markers without assigning hierarchical weight, PIM2 and lactate remained statistically significant predictors (*p* < 0.01), whereas the Pediatric Trauma Score did not contribute additional prognostic value (*p* = 0.75). Results from the univariable and multivariable logistic regression analyses are presented in Table 2.

**Table 2:** Simple and multivariate logistic regression of prognostic markers in relation to the outcome of mortality.

Variable	B	SE	df	p-value	OR (Exp[B])	95 % CI for OR
**Simple Binary Logistic Regression**						
**Log10(PIM2)**	3.585	0.437	1	<0.001	36.07	15.30–85.01
**Log10(lactate)**	5.424	0.839	1	<0.001	226.75	43.73–1174.91
**PTS**	−0.425	0.064	1	<0.001	0.65	0.57–0.74
**Binary Multivariate Logistic Regression**						
**Step 1** **Log10(PIM2)**	3.235	0.467	1	<0.001	25.40	10.16–63.51
**Step 2** **Log10(PIM2)** **Log10(lactate)**	2.516 2.927	0.487 0.957	1	<0.001 0.002	12.37 18.68	4.76–32.18 2.86–121.83

PIM2, Pediatric Index of Mortality 2; PTS, Pediatric Trauma Score; B, regression coefficient; SE, standard error; OR, odds ratio; CI, confidence interval; df, degrees of freedom.

## Discussion

To our knowledge, this study presents the largest case series of pediatric patients admitted to a trauma PICU in Brazil, and one of the largest in Latin America. Approximately 60 % of the children were injured in their own homes. Most of the patients who did not survive their PICU stay had not received pre-hospital care and arrived at the PICU in critical condition. Individually, the Pediatric Index of Mortality 2, lactate levels, and the Pediatric Trauma Score were reliable predictors of mortality in this population. However, when simultaneously included in a multivariate logistic regression model, the PTS did not significantly improve the model's predictive performance.

Unlike general-population mortality statistics such as those reported by the World Health Organization (WHO), the present data reflect a specific population of trauma patients who survived the initial event and were admitted alive to the PICU. The overall case-fatality in the case series was 1.5 %. Domestic accidents were the main cause of admission, with burns (including scalds and flame injuries) accounting for 32.6 % of all cases. This is likely attributable to our institution’s role as the designated referral center for pediatric burn care in the state, leading to a significant influx of such cases from other centers. In contrast, types of trauma typically associated with higher mortality were less common in the present sample. Drowning accounted for 1.9 % of admissions but represented 31.8 % of all deaths, while suicide attempts comprised 1.3 % of admissions and 9.1 % of overall mortality, with all deaths in this group occurring among adolescents. A potential explanation is that these types of injuries often do not require care at specialized trauma centers and are instead managed at general PICUs closer to the site of occurrence.

The high prevalence of injuries occurring within the home environment is not a new phenomenon in Brazil. In this case series, approximately 60 % of pediatric trauma patients admitted to the PICU sustained their injuries at home, with the majority of these cases involving children under 6 years of age. A 2015 multicenter study involving emergency departments from major Brazilian cities reported that 67 % of injuries occurred in the home setting, a proportion that rose to 88 % among children under one year of age [8]. Several risk factors for unintentional injuries in children under 4 years of age remain prevalent in Brazil, including having an adolescent mother, low maternal schooling, and low family socioeconomic status [9]. In addition, inadequate supervision and developmental characteristics intrinsic to early childhood — such as intense curiosity, a strong tendency to imitate adults, and the inability to foresee and avoid danger—likely contribute to the high incidence of home-related injuries in this age group [8].

Only 29 patients (1.9 %) in our case series sustained injuries at school, a low proportion that has also been reported in previous studies [10,11]. Although the underlying causes cannot be confirmed from our data or from those studies, this may reflect both improved school safety measures and a global trend toward more risk-averse play. Some authors advocate for “risky play” as beneficial to child development, distinguishing it from hazards, but the authors were unable to explore this hypothesis further [12].

Based on the characteristics of the present sample and the outcomes observed, the authors can, as a conceptual exercise, infer a profile of the non-surviving patient. This profile would include: (1) children sustaining trauma either at home or in traffic-related incidents, such as motor vehicle accidents or pedestrian injuries; (2) not receiving care at the trauma scene; (3) arriving at the PICU with elevated PIM2 scores and serum lactate levels, along with low PTS and Glasgow Coma Scale (GCS) scores; (4) requiring mechanical ventilation and vasoactive support; and (5) dying after a short PICU stay, most commonly progressing to brain death. This hypothetical profile may assist clinicians in the early identification of patients at the highest risk of poor outcomes.

The main practical implication of the present study, beyond characterizing the profile of pediatric patients admitted to a specialized trauma PICU in Brazil, was to confirm the prognostic value of PIM2, lactate, and the Pediatric Trauma Score, both individually and in combination [13, 14, 15]. These markers are simple and widely accessible across most PICUs, reinforcing their clinical applicability. Notably, in the case series, PTS did not enhance mortality prediction when used alongside PIM2 and lactate, which underscores its primary role in prehospital triage and exposes its limitations once patients are stabilized and receiving intensive care. The PTS was developed in the 1980s and has proven useful for identifying severely injured children at high risk of mortality during initial assessment [4].

The PIM2 score was developed for general pediatric ICU populations; however, it has proven useful in specific subgroups, including trauma, sepsis, and surgical conditions [15, 16, 17]. To date, the combined use of PIM2 and lactate as predictors of mortality in pediatric trauma has not been studied in a large case series. In the present study, the authors were able to perform this analysis in 620 patients (41 % of all patients) and demonstrated that the addition of lactate improved the already good discriminative ability of PIM2 for predicting mortality. Morris and colleagues examined this association in 2012 in a cohort of 2380 general PICU patients in the United Kingdom, reporting similar findings [18]. The present findings support further evaluation of lactate as a complementary marker in larger multicenter datasets.

When interpreting the results of the present analysis, it is important to keep in mind that the observed mortality rate of 1.5 % was low compared to the literature [10,13,15,18]. Several explanations can be proposed. First, only 17.3 % of all patients received prehospital care at the trauma scene. A study conducted in 2013 assessing the prevalence of prehospital care in motor vehicle accidents in Brazil demonstrated that only 13 % of victims received such care [19]. This underscores that access to prehospital care remains unavailable to many patients in the studied country. It is possible that many severely injured patients arrived in critical condition at the site of initial care and died before being referred to the PICU. Second, all members of our PICU team are board-certified intensivists with extensive experience in trauma care, which is not the standard across other regions of Brazil. Finally, 7 % of the patients were later transferred to other institutions for more complex procedures or for the management of chronic conditions. Mortality outcomes for these transferred patients could not be obtained.

The present study has some limitations that need to be addressed. This was a single-center study, and the authors were aware of its inherent limitations. However, given the scarcity of studies in this area, the authors considered it essential to contribute to the current evidence by publishing our findings to stimulate multicenter research efforts. As previously explained, the mortality rate was low, which limited more in-depth analyses within subgroups. The authors were able to obtain all three prognostic markers (PIM2, serum lactate, and PTS) in 620 patients. All patients who died had data available for all three markers; however, many low-severity patients did not have serum lactate collected, meaning that prognostic accuracy was evaluated only in a selected high-risk subgroup, thereby limiting external validity. Additionally, lactate was analyzed at a single time point, and the authors were unable to assess its clearance or trend throughout the PICU stay. Finally, the authors chose not to define cutoff values for the prognostic markers. These thresholds are highly influenced by the type of illness and the level of care provided. Given the single-center nature of the present study and the considerable heterogeneity in trauma etiologies among the studied patients, the authors believe that establishing cutoffs would result in imprecise analyses with limited external validity.

By providing one of the largest patient case series from Latin America in this context, the authors contribute valuable evidence to an underexplored area. The present study demonstrates that PIM2 and serum lactate are strong and independent predictors of mortality in pediatric trauma patients admitted to a specialized PICU, whereas the Pediatric Trauma Score has limited utility once the patient is already receiving intensive care support. These findings support the use of simple, widely available markers for early risk stratification and guided clinical decision-making in pediatric trauma care.

## Data availability

The data that support the findings of this study are available from the corresponding author.

## Funding

This study was financed in part by the *Coordenação de Aperfeiçoamento de Pessoal de Nível Superior – Brasil* (CAPES) – Finance Code 001.

## Conflicts of interest

Dr. Vieira disclosed that this study was financed in part by the *Coordenação de Aperfeiçoamento de Pessoal de Nível Superior – Brasil* (CAPES) – Finance Code 001. The remaining authors have disclosed that they do not have any potential conflicts of interest.

## Appendix Supplementary materials

Image, application 1
