# Supplementary material for: PIM2, lactate, and trauma score to predict mortality in critically ill pediatric trauma patients
Source: J Pediatr (Rio J). 2026 Feb 17;102(2):101509. doi: 10.1016/j.jped.2026.101509 (PMC12926633; doi:10.1016/j.jped.2026.101509)
Supplement: Supplementary file 1 [file mmc1.docx]

**JPED-D-25-00413_ Supplementary Material**

**Supplemental Material 1 Table.** Demographics, Trauma Profiles, and Outcomes Across Pediatric Age Groups in Trauma ICU Admissions Over a 7-Year Period

| **Variable** | **0-6 years (n=894)** | **6-12 years**  **(n=407)** | **Over 12 years**  **(n=194)** | **p-value*** |
| --- | --- | --- | --- | --- |
| **Sex, n (%)**  Male  Female | 519 (58.1)^a^  375 (41.9)^a^ | 263 (64.6)^a,b^  144 (35.4)^a,b^ | 138 (71.1)^b^  56 (29.9)^b^ | <0.01 |
| **Year of injury, n (%)**  2018  2019  2020  2021  2022  2023  2024  2025 | 112 (12.5)  150 (16.8)  124 (13.9)  135 (15.1)  111 (12.4)  117 (13.1)^a^  132 (14.8)  13 (1.5) | 59 (14.5)  45 (11.1)  54 (13.3)  63 (15.5)  54 (13.3)  64 (15.7)^a,b^  59 (14.5)  9 (2.2) | 19 (9.8)  20 (10.3)  19 (9.8)  22 (11.3)  36 (18.6)  40 (20.6)^b^  34 (17.5)  4 (2.1) | 0.01 |
| **Season, n (%)**  Summer  Autumn  Winter  Spring | 236 (26.4)  217 (24.3)  215 (24)  226 (25.3) | 118 (29)  92 (22.6)  92 (22.6)  105 (25.8) | 47 (24.2)  52 (26.8)  52 (26.8)  43 (22.2) | 0.68 |
| **City of origin, n (%)**  Capital  Metropolitan Region  Contryside | 220 (24.6)^a^  389 (43.5)^a^  285 (31.9) | 102 (25.1)^a^  166 (40.8)^a,b^  139 (34.2) | 78 (40.2)^b^  60 (30.9)^b^  56 (28.9) | <0.01 |
| **Time of injury, n (%)**  0-5:59 am  6-11:59 am  12-5:59 pm  6-11:59 pm  Unknown | 38 (4.3)  165 (18.5)^a^  344 (38.5)^a^  325 (36.4)  22 (2.5) | 14 (3.4)  68 (16.7)^a,b^  188 (46.2)^b^  129 (31.7)  8 (2) | 15 (7.7)  20 (10.3)^b^  97 (50)^b^  58 (29.9)  4 (2.1) | <0.01 |
| **Location of trauma, n (%)**  Home  Relative´s or friend´s home  School  Public roads  Other  Unknown or not applicable | 672 (75.2)^a^  48 (5.4)  9 (1)^a^  132 (14.8)^a^  19 (2.1)^a^  14 (1.6) | 152 (37.3)^b^  23 (5.7)  16 (3.9)^b^  193 (47.4)^b^  20 (4.9)^b^  3 (0.7) | 63 (32.5)^b^  5 (2.6)  4 (2.1)a,b  100 (51.5)^b^  17 (8.8)^b^  5 (2.6) | <0.01 |
| **Prehospital care, n (%)**  Yes  No  Unknown | 73 (8.2)^a^  780 (87.2)^a^  41 (4.6) | 98 (24.1)^b^  285 (70)^b^  24 (5.9) | 87 (44.8)^c^  95 (49)^c^  12 (6.2) | <0.01 |
| **Pediatric Trauma Score, md (IQR)** | 8 (7-10) | 9 (6-10) | 8 (4-10) | <0.01 |
| **PIM 2 %, md (IQR)** | 0.8 (0.8-1.1) | 0.8 (0.8-1.2) | 0.9 (0.8-3.5) | <0.01 |
| **Lactate in mmol/L, md (IQR)** | 1.7 (1.1-2.4) | 1.7 (1.1-2.6) | 1.8 (1.27-2.7) | 0.30 |
| **Pediatric Trauma Score <8, n (%)**  Yes  No | 288 (32.2)^a^  606 (67.8)^a^ | 132 (32.4)^a,b^  275 (67.6)^a,b^ | 81 (41.8)^b^  113 (58.2)^b^ | 0.03 |
| **GCS, md (IQR)^1^** | 15 (14-15) | 15 (14-15) | 14 (11-15) | <0.01 |
| **GCS by category, n (%)^2^**  <=8  9-12  13-15  Not measurable | 21 (6.5)  16 (4.9)  252 (77.5)^a^  36 (11.1)^a^ | 15 (7.7)  16 (8.2)  136 (70.1)^a,b^  27 (13.9)^a,b^ | 10 (11.9)  6 (7.1)  49 (58.3)^b^  19 (22.6)^b^ | 0.02 |
| **Type of Trauma, n (%)**  Pedestrian struck  Motor Vehicle collision  Fall from height  Gunshot wound  Assalt  Scald injury  Burn (flame)  Eletric shock  Bicycle fall  Bite or scratch  Venomous animal injury (including spider)  Exogenous poisonig  Object falling on the child  Drowning  Horse kick  Sharp force injury  Hanging  Crush injury  Chemical burn  Foreign body aspiration  Others  Unknown | 58 (6.5)^a^  58 (6.5)^a^  207 (23.2)^a^  12 (1.3)^a^  8 (0.9)^a^  327 (36.6)^a^  57 (6.4)^a^  12 (1.3)  9 (1)^a^  15 (1.7)  11 (1.2)  22 (2.5)^a^  32 (3.6)  24 (2.7)^a^  9 (1)  3 (0.3)^a^  0 (0)^a^  2 (0.2)  2 (0.2)  2 (0.2)  18 (2)  6 (0.7) | 87 (21.4)^b^  53 (13)^b^  87 (21.4)^a,b^  11 (2.7)^a^  5 (1.2)^a,b^  32 (7.9)^b^  45 (11.1)^b^  7 (1.7)  28 (6.9)^b^  7 (1.7)  4 (1)  0 (0)^b^  14 (3.4)  2 (0.5)^b^  8 (2)  2 (0.5)^a,b^  0 (0)^a^  1 (0.2)  1 (0.2)  2 (0.5)  11 (2.7)  0 (0) | 38 (19.6)^b^  31 (16)^b^  27 (13.9)^b^  14 (7.2)^b^  7 (3.6)^b^  6 (3.1)^b^  20 (10.3)^a,b^  3 (1.5)  14 (7.2)^b^  0 (0)  1 (0.5)  12 (6.2)^c^  3 (1.5)  3 (1.5)^a,b^  1 (0.5)  4 (2.1)^b^  5 (2.6)^b^  0 (0)  0 (0)  0 (0)  3 (1.5)  2 (1) | <0.01 |
| **Main injury type, n (%)**  Traumatic brain injury  Facial trauma  Spinal cord injury  Thoracic trauma  Hapatic abdominal trauma  Splenic abdominal trauma  Renal abdominal trauma  Hollow abdominal trauma  Pancreatic abdominal trauma  Genitourinary trauma  Other abdominal trauma  Skin trauma or scalping  Musculoskeletal trauma  Burn  Drowning  Exogenous poisonig  Venomous animal injury (including spider)  Eletric shock  Hypoxic-ischemic injury  Medical (non-trauma) admission  Ocular trauma  Pelvic trauma  Others | 313 (35)^a^  19 (2.1)  3 (0.3)  12 (1.3)^a^  12 (1.3)  10 (1.1)^a^  2 (0.2)^a^  3 (0.3)^a^  3 (0.3)  1 (0.1)^a^  1 (0.1)^a^  4 (0.4)  40 (4.5)^a^  396 (44.3)^a^  10 (1.1)  22 (2.5)^a^  11 (1.2)  2 (0.2)  15 (1.7)  3 (0.3)^a,b^  6 (0.7)  0 (0)^a^  6 (0.7) | 179 (44)^b^  15 (3.7)  1 (0.2)  19 (4.7)^b^  9 (2.2)  7 (1.7)^a,b^  3 (0.7)^a,b^  11 (2.7)^b^  6 (1.5)  6 (1.5)^b^  5 (1.2)^b^  2 (0.5)  42 (10.3)^b^  82 (20.1)^b^  2 (0.5)  0 (0)^b^  5 (1.2)  1 (0.2)  3 (0.7)  0 (0)^b^  5 (1.2)  1 (0.2)^a,b^  3 (0.7) | 76 (39.2)^a,b^  8 (4.1)  0 (0)  9 (4.6)^b^  6 (3.1)  10 (5.2)^b^  5 (2.6)^b^  4 (2.1)^b^  1 (0.5)  3 (1.5)^b^  1 (0.5)^a,b^  1 (0.5)  13 (6.7)^a,b^  27 (13.9)^b^  3 (1.5)  12 (6.2)^c^  1 (0.5)  2 (1)  6 (3.1)  3 (1.5)^a^  0 (0)  2 (1)^b^  1 (0.5) | <0.01 |
| **Suicide attempt**  Yes  No | 0 (0)^a^  894 (100)^a^ | 0 (0)^a^  407 (100)^a^ | 19 (9.8)^b^  175 (90.2)^b^ | <0.01 |
| **Suspected non-accidental trauma**  Yes  No  Unknown | 22 (2.5)  871 (97.4)  1 (0.1) | 6 (1.5)  400 (98.3)  1 (0.2) | 4 (2.1)  190 (97.9)  0 (0) | 0.74 |
| **Mechanical ventilation**  Yes  No | 133 (14.9)^a^  761 (85.1)^a^ | 83 (20.4)^b^  324 (79.6)^b^ | 63 (32.5)^b^  131 (67.5)^c^ | <0.01 |
| **Ventilatior-free days, md (IQR)^3^** | 25 (21-27) | 25 (21-27) | 26 (19.75-27) | 0.88 |
| **Vasoactive drugs**  Yes  No | 63 (7)^a^  831 (93)^a^ | 48 (11.8)^b^  359 (88.2)^b^ | 34 (17.5)^b^  160 (82.5)^b^ | <0.01 |
| **PICU length of stay in days, md (IQR)** | 4 (2-10) | 4 (2-10) | 5.50 (3-10.25) | 0.21 |
| **PICU stay > 7 days, n (%)**  Yes  No | 302 (33.8)  592 (66.2) | 116 (28.5)  291 (71.5) | 66 (34)  128 (66) | 0.14 |
| **Hospital length of stay in days, md (IQR)** | 7 (3-15) | 6 (3-13) | 7 (4-14.25) | 0.31 |
| **Hospital stay > 14 days, n (%)**  Yes  No | 228 (25.5)  666 (74.5) | 95 (23.3)  312 (76.7) | 48 (24.7)  146 (75.3) | 0.70 |
| **Complicated outcome^4^, n (%)**  Yes  No | 71 (7.9)^a^  823 (92.1)^a^ | 49 (12)^a,b^  358 (88)^a,b^ | 37 (19.1)^b^  157 (80.9)^b^ | <0.01 |
| **Death, n (%)**  No  Yes | 880 (98.4)  14 (1.6) | 403 (99)  4 (1) | 190 (97.9)  4 (2.1) | 0.55 |
| **Discharge disposition, n (%)**  Ward  Home  Transfer to another hospital  Shelter or institution  Death | 389 (43.5)  442 (49.4)  47 (5.3)^a^  2 (0.2)  14 (1.6) | 186 (45.7)  185 (45.5)  30 (7.4)^a^  2 (0.5)  4 (1) | 76 (39.2)  86 (44.3)  28 (14.4)^b^  0 (0)  4 (2.1) | <0.01 |

PICU, Pediatric Intensive Care Unit; PIM2, Pediatric Index of Mortality 2; GCS, Glasgow Coma Scale; md, median; IQR, interquartile range.

^a,b,c^ Same letter indicates no significant difference between groups. Superscript letters are only displayed when differences between groups are statistically significant.
^1^ Data available for 521 patients.Complicated outcome includes patients who died, required mechanical ventilation for more than 7 days, or received vasoactive drugs.
^2^ Data available for 603 patients.Ventilator-free days = 28 – (days on MV). A value of zero was assigned to patients who died.
^3^ Ventilator-free days = 28 – (days on MV). A value of zero was assigned to patients who died.
^4^ Complicated outcome includes patients who died, required mechanical ventilation for more than 7 days, or received vasoactive drugs.
*Considered statistically significant if p < 0.05. Chi-square or Fisher’s exact test was used for categorical variables, as appropriate. For non-parametric continuous variables, the Mann-Whitney U test was applied. The Bonferroni correction was applied to adjust for multiple comparisons.
